# Predicting solute transfer rate in patients initiating peritoneal dialysis

**DOI:** 10.1007/s40620-023-01862-y

**Published:** 2024-01-30

**Authors:** David A. Jaques, Andrew Davenport

**Affiliations:** 1 Division of Nephrology, Geneva University Hospitals, Rue Gabrielle-Perret-Gentil 4, 1205 Geneva, Switzerland; 2 UCL Department of Nephrology, Royal Free Hospital, University College London, London, UK

**Keywords:** Peritoneal dialysis, Peritoneal solute transfer rate, Fast transporter, Peritoneal equilibration test

## Abstract

**Background:**

While assessment of membrane characteristics is fundamental to peritoneal dialysis (PD) prescription in patients initiating therapy, peritoneal equilibration test has theoretical and practical drawbacks. We wished to determine whether an equation using simple clinical variables could predict fast (above population mean) peritoneal solute transfer rate without dialysate sampling.

**Methods:**

We measured peritoneal solute transfer rate, as determined by peritoneal equilibration test using the 4-h dialysate to plasma creatinine ratio, in consecutive PD outpatients attending a single tertiary hospital for their first clinical follow-up within 3 months of dialysis initiation. An equation estimating peritoneal solute transfer rate based on readily available clinical variables was generated in a randomly selected modeling group and tested in a distinct validation group.

**Results:**

We included 712 patients, with 562 in the modeling group and 150 in the validation group. Mean age was 58.4 ± 15.9 with 431 (60.5%) men. Mean peritoneal solute transfer rate value was 0.73 ± 0.13. An equation based on gender, race, serum sodium and albumin yielded a receiving operator characteristics (ROC) area under the curve (AUC) to detect fast peritoneal solute transfer rate (> 0.73) of 0.74 (0.67–0.82). Estimated peritoneal solute transfer rate values based on percentiles 15th (> 0.66), 20th (> 0.68), 25th (> 0.69) and 30th (> 0.70) could rule out fast peritoneal solute transfer rate with negative predictive values of 100%, 93.5%, 84.2% and 80.0%, respectively.

**Conclusions:**

An equation based on simple clinical variables allows ruling out fast transport in a significant proportion of patients initiating PD with a high degree of confidence without requiring dialysate sampling. This could prove useful in guiding dialysis prescription of PD patients in daily clinical practice, particularly in low-resource settings.

**Graphical abstract:**

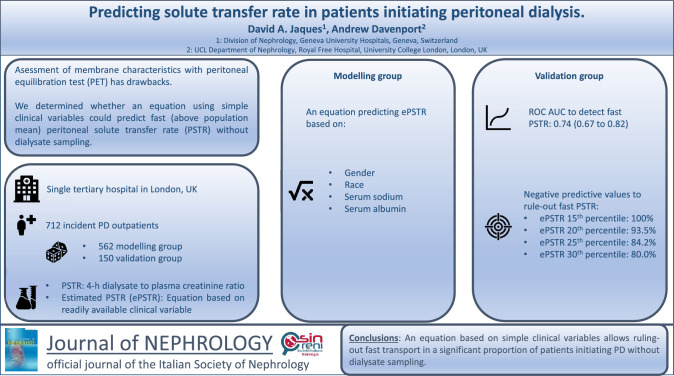

## Introduction

Peritoneal dialysis (PD) uses the peritoneum as a semi-permeable membrane in order to achieve solute clearance as well as ultrafiltration in patients with end-stage kidney disease (ESKD). It is well-known that variation in peritoneal membrane function is high, both among, as well as within, individuals over time [1]. Diffusive transport characteristics of the peritoneum are best determined using the peritoneal equilibration test consisting in a standardized 4-h dwell with concomitant blood and dialysate electrolyte measurement. Based on the 4-h dialysate to plasma creatinine ratio, peritoneal equilibration test allows objective characterization of the peritoneal solute transfer rate. Prior guidelines used pre-defined peritoneal solute transfer rate cut-offs to define discrete transport categories labeled as “slow” (peritoneal solute transfer rate < 0.55), “average” (peritoneal solute transfer rate ≥ 0.55 and ≤ 0.80) or “fast” (peritoneal solute transfer rate > 0.80) [2]. Most recent international guidelines however abolished such categorization as absolute peritoneal solute transfer rate values show marked variability between centers, thus preventing meaningful harmonization [3]. Nevertheless, assessment of peritoneal membrane characteristics is important to PD prescription in incident patients starting dialysis as this could inform prognosis and potentially guide dialytic treatment. Notably, a fast peritoneal solute transfer rate is associated with an increased risk of mortality and hospitalization, likely explained by poor ultrafiltration [4]. Tailoring initial prescription based on measured peritoneal solute transfer rate could partly mitigate this risk [5, 6]. Consequently, the latest international guidelines recommend that peritoneal equilibration test be conducted early in the course of dialysis treatment (between 6 and 12 weeks after initiation) [3]. Later in the course of treatment, as membrane characteristics tend to change over time, transport characteristics should be evaluated regularly as well as when clinically indicated to ensure that prescription matches the patient’s specificities [2, 3]. As discrete categorization has been abandoned, fast transport is now simply defined as a peritoneal solute transfer rate value above the population mean. Formal peritoneal equilibration test assessment is however not without limitations, including difficult standardization, inaccurate measurements, nursing burden and economic cost [7, 8]. When initiating PD, tailoring prescription is further complicated by the fact that peritoneal transport characteristics are notably difficult to predict in individual patients with controversial data regarding potential determinants and significant variations among various studied populations [9]. Thus, given the valuable information provided by peritoneal solute transfer rate in managing PD patients in routine practice, the latest guidelines recommended that new biomarkers be developed to predict, identify and monitor peritoneal membrane function [3].

In today’s clinical practice, physicians are lacking simple tools to rapidly characterize membrane characteristics and inform treatment decisions in incident PD patients starting dialysis. Consequently, we wished to conduct a retrospective study in order to (i) identify determinants of peritoneal solute transfer rate at dialysis initiation and (ii) assess the clinical performance of an equation based on readily available variables in predicting peritoneal solute transfer rate without relying on peritoneal equilibration test.

## Methods

### Participants and study design

We consecutively included PD incident outpatients who recently started dialysis and attended a single tertiary hospital for their first clinical follow-up and assessment of peritoneal membrane function between 2006 and 2022. The timeframe of this first assessment was restricted to the first 3 months following dialysis initiation in agreement with the latest international guidelines [3]. Patients were treated either with continuous ambulatory PD (CAPD) or automated PD (APD) using a cycler. Icodextrin solution could be used but no patient was prescribed a glucose dialysate concentration above 2.27%. Exclusion criteria were (i) dialysis vintage > 3 months and (ii) peritonitis or emergency admission to hospital in the prior 3 months.

### Collection of variables

Peritoneal solute transfer rate was determined using peritoneal equilibration test at the end of a 4-h dwell with 2 L of 2.27% dialysate solution. To avoid interference from a prior icodextrin dwell and standardize glucose exposure, all patients arrived with a 2.27% dwell instilled prior to peritoneal equilibration test. Fast peritoneal solute transfer rate was defined as peritoneal solute transfer rate above the population mean according to the latest guidelines [3]. Dialysis adequacy and residual kidney function were based on clearance of small molecules and expressed as Kt/V as calculated by standard methods from 24-h urine collections and samples from all spent dialysates. Weight was defined as actual body weight at the time of clinical evaluation. Body surface area was calculated using the Haycock formula and expressed as m^2^. Body mass index (BMI) was expressed as kg/m^2^. Blood pressure was recorded in the supine position after the patient had drained out dialysate and rested for a minimum of 30 min (Dinamap, Critikon Corporation, Tampa, FL, USA). Serum creatinine and urea were measured by a standard biochemical and enzymatic methodology (Roche Modular P, Roche Diagnostics, Lewes, UK). Serum albumin was measured by the bromocresol green method (Roche Modular P, Roche Diagnostics, Lewes, UK). Dialysate creatinine was measured enzymatically to avoid interference of glucose on Jaffe method [10]. All laboratories were UK accredited. Extracellular water to total body water ratio was measured by multifrequency bioelectrical impedance using a standardized protocol (InBody 720, Seoul, South Korea), with dialysate drained out [11]. Hypertension and diabetes were defined based on the presence of related medications. Physicians in charge had unrestricted access to collected variables during follow-up.

### Statistical analysis

Continuous variables are expressed as mean and standard deviation (SD) or median and interquartile range (IQR) according to distribution. Categorical variables are expressed as number and relative frequencies. Normality of distribution was assessed graphically. Variables were compared between groups using Student’s *t* test and Chi-square for continuous and categorical variables, respectively. For regression models, normality of residuals and homoscedasticity of residuals were assessed graphically. Results are presented as β coefficients and associated 95% confidence intervals (95% CI) as well as p-values. A two-sided *p* value < 0.05 was considered significant throughout. Statistical analyses were conducted using STATA version 17 (StataCorp, 4905 Lakeway Drive, College Station, Texas 77845 USA).

### Predictors of peritoneal solute transfer rate

A multivariate linear regression model was used with peritoneal solute transfer rate as the dependent variable and the following a priori selected independent variables: Age, gender, race, BMI, body surface area, diabetes, Davies comorbidity score, dialysis vintage, systolic blood pressure, diastolic blood pressure, serum glucose, C-reactive protein, hemoglobin, serum sodium, serum albumin, serum creatinine, serum urea, daily urine output and extracellular water/total body water. A backward stepwise procedure was applied, sequentially keeping only independent variables with *p* values < 0.05 in the final model. In order to allow direct comparison of relative effect sizes of significant predictors, continuous variables were standardized to a mean of 0 and a SD of 1 in regression models.

### Generation of predictive equation for peritoneal solute transfer rate

Patients were randomly divided into two groups; a modeling group used to construct a predictive equation estimating peritoneal solute transfer rate and a validation group used to assess equation performances to predict measured peritoneal solute transfer rate. The number of patients in the validation was empirically pre-specified at 150. The predictive equation was generated using the multivariate fractional polynomial algorithm. Briefly, the multivariate fractional polynomial algorithm allows backward elimination of possible predictors and selection of a fractional polynomial function accounting for the potential non-linear relationship of continuous variables. To avoid overfitting, allowed powers were 0 (corresponding to log-transform by definition), 1 and 2. To obtain a reasonably parsimonious model, *p* value thresholds for inclusion of covariates and determination of significance of fractional polynomial transformations were both specified at 0.05. In generating the predictive equation, the following a priori potential predictors were considered: Age, gender, race, BMI, body surface area, diabetes, Davies comorbidity score, dialysis vintage, serum glucose, C-reactive protein, hemoglobin, serum sodium, serum albumin, serum creatinine as well as serum urea.

### Assessment of predictive equation for peritoneal solute transfer rate

Performance of the predictive equation for estimated peritoneal solute transfer rate was measured in the validation group. Correlation between peritoneal solute transfer rate and estimated peritoneal solute transfer rate values was assessed with Spearman’s and Pearson’s correlation coefficients. Agreement between peritoneal solute transfer rate and estimated peritoneal solute transfer rate was assessed using Bland and Altman analysis. Bias was defined as the median of the difference between peritoneal solute transfer rate and estimated peritoneal solute transfer rate. Receiving operator characteristics (ROC) analyses were performed using different cut-off values of estimated peritoneal solute transfer rate. Finally, sensitivity, specificity, positive as well as negative predictive values were computed for various cut-offs.

## Results

The entire study cohort consisted of 712 patients with 562 randomly attributed to the modeling group and 150 to the validation group. Mean age was 58.4 ± 15.9 and 431 (60.5%) were men. Median dialysis vintage was 2 (2–3) months. Mean peritoneal solute transfer rate value was 0.73 ± 0.13 and the number of patients with fast peritoneal solute transfer rate (> 0.73) was 375 (52.6%). Patient’s characteristics according to random grouping are described in Table 1. The proportion of male patients was higher in the modeling group as compared to the validation group. Other characteristics were similar between groups.

**Table 1:** Patient characteristics

Characteristic	Overall (*N* = 712)	Modeling group (*N* = 562)	Validation group (*N* = 150)	*p* value
Clinical characteristics				
Age (years)	58.4 ± 15.9	58.6 ± 16.2	57.7 ± 14.9	0.531
Gender (male)	431 (60.5%)	356 (63.3%)	75 (50.0%)	**0.003**
Race (White)	331 (46.4%)	256 (45.5%)	75 (50.0%)	0.332
BMI (kg/m^2^)	26.5 ± 5.0	26.4 ± 4.9	26.7 ± 5.4	0.545
BSA (m^2^)	1.86 ± 0.24	1.86 ± 0.24	1.86 ± 0.25	0.956
Diabetes	304 (42.7%)	243 (43.2%)	61 (40.6%)	0.572
Hypertension	584 (82.0%)	462 (82.2%)	122 (81.3%)	0.805
Davies Score	1 (0–2)	1 (0–2)	1 (0–2)	0.718
SBP (mmHg)	140.2 ± 23.4	140.5 ± 24.0	138.9 ± 21.2	0.459
DBP (mmHg)	81.3 ± 14.8	81.2 ± 15.0	81.6 ± 14.1	0.766
Laboratory characteristics				
Hemoglobin (g/L)	110.0 ± 15.5	109.7 ± 14.7	111.4 ± 18.0	0.225
Albumin (g/L)	36.7 ± 4.8	36.6 ± 4.7	36.9 ± 4.9	0.499
CRP (mg/L)	4 (1–9)	4 (2–9)	3 (1–9)	0.706
Serum calcium (mmol/L)	2.32 ± 0.17	2.32 ± 0.17	2.31 ± 0.17	0.670
Serum phosphate (mmol/L)	1.54 ± 0.41	1.53 ± 0.41	1.57 ± 0.41	0.277
PTH (pmol/L)	25.5 (15.0–41.0)	25.8 (14.9–42.8)	23.1 (15.5–38.4)	0.071
Dialysis characteristics				
Vintage (months)	2 (2–3)	2 (2–3)	2 (2–3)	0.568
PD mode (APD)	491 (69.7%)	384 (69.1%)	107 (71.8%)	0.536
Dwell volume (L)	1.78 ± 0.33	1.78 ± 0.35	1.78 ± 0.26	0.854
Number of cycles	4.8 ± 2.1	4.8 ± 2.1	4.9 ± 2.0	0.854
PSTR	0.73 ± 0.13	0.73 ± 0.13	0.72 ± 0.14	0.374
Fast PSTR	375 (52.6%)	301 (53.5%)	74 (49.3%)	0.357
KtV PD	1.05 + /0.43	1.05 ± 0.43	1.04 ± 0.41	0.883
KtV urine	1.45 ± 0.99	1.46 ± 1.03	1.39 ± 0.82	0.463
Daily urine output (mL/day)	1′259 ± 808	1′272 ± 829	1′210 ± 724	0.402

Results presented as mean ± SD or median (IQR) according to distribution

Bold values correspond to *p* < 0.05

*BMI* body mass index, *BSA* body surface area, *SBP* systolic blood pressure, *DBP* diastolic blood pressure, *CRP* C reactive protein, *PTH* parathormone, *PD* peritoneal dialysis, *APD* automated PD, *PSTR* peritoneal solute transfer rate, *SD* standard deviation, *IQR* interquartile range

### Predictors of peritoneal solute transfer rate

Multivariate analyses on the entire study cohort included 637 patients without missing values on considered covariates. In the final multivariate model, by order of decreasing effect sizes, factors positively associated with peritoneal solute transfer rate were (Table 2): Male gender, systolic blood pressure, extracellular water/total body water, serum urea and dialysis vintage. By order of decreasing effect sizes, factors negatively associated with peritoneal solute transfer rate were: Serum sodium, albumin, glucose and creatinine. Adjusted R2 metric for the final model was 25.2%. Variables not associated with peritoneal solute transfer rate in the final model were successively discarded in the following order during the backward stepwise procedure: Daily urine output (*p* = 0.999), C-reactive protein (*p* = 0.626), diabetes (*p* = 0.450), Davies comorbidity score (*p* = 0.581), hemoglobin (*p* = 0.333), diastolic blood pressure (*p* = 0.230), race (*p* = 0.130), BMI (*p* = 0.129), body surface area (*p* = 0.531) and age (*p* = 0.068).

**Table 2:** Predictors of peritoneal solute transfer rate

Variable	β	95% CI	*p* value
Gender (male)	0.24	0.09 to 0.38	**0.001**
SBP (mmHg)	0.17	0.10 to 0.24	** < 0.001**
ECW/TBW	0.15	0.07 to 0.24	** < 0.001**
Urea (mmol/L)	0.09	0.01 to 0.16	**0.013**
Dialysis vintage (months)	0.06	0.00 to 0.13	**0.049**
Sodium (mmol/L)	− 0.27	− 0.34 to − 0.19	** < 0.001**
Albumin (g/L)	− 0.22	− 0.30 to − 0.14	** < 0.001**
Glucose (mmol/L)	− 0.11	− 0.18 to − 0.03	**0.003**
Creatinine (umol/L)	− 0.07	− 0.15 to − 0.00	**0.042**

All continuous variables were standardized to a mean of 0 and a SD of 1

Bold values correspond to *p* < 0.05

*PSTR* peritoneal solute transfer rate, *SBP* systolic blood pressure, *ECW/TBW* extracellular water to total body water ratio

### Generation of predictive equation for peritoneal solute transfer rate

During the multivariate fractional polynomial process, age, BMI, body surface area, diabetes, Davies comorbidity score, dialysis vintage, serum glucose, C-reactive protein, hemoglobin and serum creatinine were discarded while gender, race, serum sodium and serum albumin were included in the equation. No power transformation was applied by the multivariate fractional polynomial algorithm and the final predictive equation for estimated peritoneal solute transfer rate was given as:

$${\text{ePSTR}}=0.0288583*{\text{gender}}+0.024756*{\text{race}}-0.0081452*{\text{sodium}}-0.0082967*{\text{albumin}}+2.136475,$$

where gender is 1 for men and 0 for women and race is 1 for White and 0 for non-White. Adjusted R2 metric was 19.6% and 18.4% in the modeling and validation groups, respectively.

### Assessment of predictive equation for peritoneal solute transfer rate

**Fig. 1:**
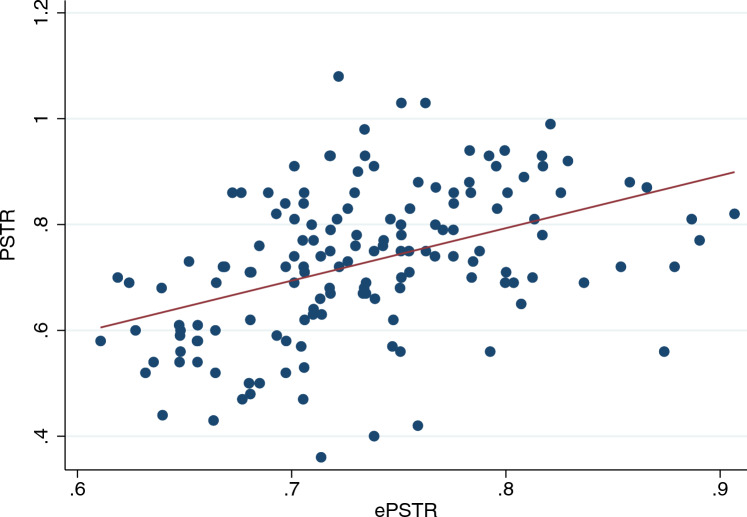
Relationship between PSTR and ePSTR in the validation group. *PSTR* peritoneal solute transfer rate, *ePSTR* estimated peritoneal solute transfer rate

**Fig. 2:**
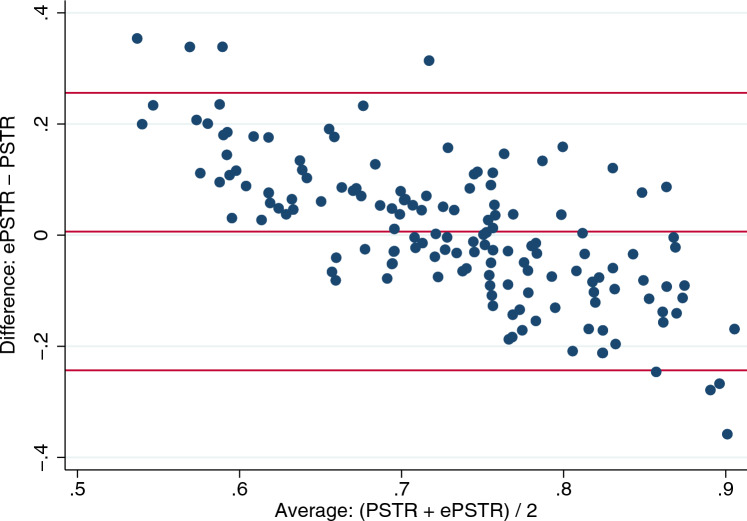
Bland and Altman plot for PSTR and ePSTR in the validation group. *PSTR* peritoneal solute transfer rate, *ePSTR* estimated peritoneal solute transfer rate

**Fig. 3:**
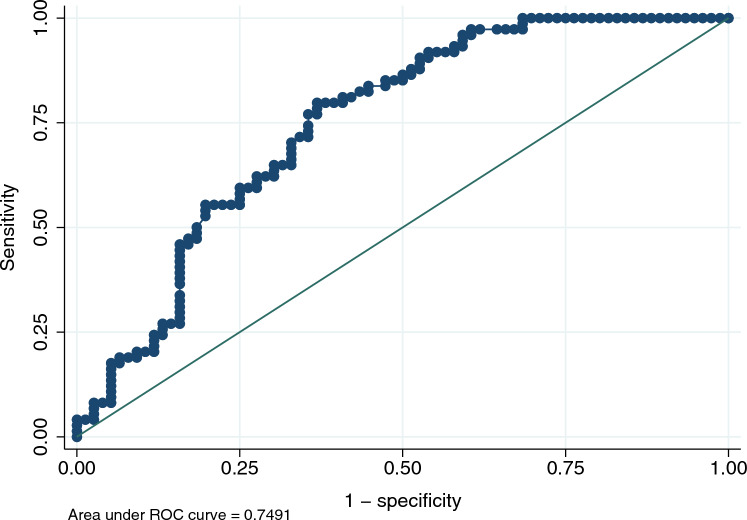
ROC curve in the validation group to detect fast PSTR (> 0.73). *ROC* receiving operator characteristics, *PSTR* peritoneal solute transfer rate

The relationship between peritoneal solute transfer rate and estimated peritoneal solute transfer rate in the validation group is depicted in Fig. 1. Spearman’s and Pearson’s coefficients were 0.48 and 0.43, respectively (*p* < 0.001). Bland and Altman analysis is depicted in Fig. 2. Bias was 0.00 (− 0.03 to 0.03) and visual inspection showed proportional bias with overestimation of peritoneal solute transfer rate in the lowest range and underestimation of peritoneal solute transfer rate in the highest range. Area under the curve (AUC) of ROC analysis to detect fast peritoneal solute transfer rate (> 0.73) was 0.749 (0.670–0.827) (Fig. 3).

Diagnostic performance of equation to predict fast peritoneal solute transfer rate (> 0.73) based on various estimated peritoneal solute transfer rate cut-offs is given in Table 3. Cut-offs are constructed as percentiles of estimated peritoneal solute transfer rate distribution. Specifically, estimated peritoneal solute transfer rate values based on percentiles 15th (> 0.66), 20th (> 0.68), 25th (> 0.69) and 30th (> 0.70) could rule out fast peritoneal solute transfer rate (> 0.73) with negative predictive values of 100%, 93.5%, 84.2% and 80.0%, respectively.

**Table 3:** Diagnostic performance of equation to predict fast peritoneal solute transfer rate (> 0.73) based on various estimated peritoneal solute transfer rate cut-offs

Status to be identified	AUC	95% CI	ePSTR cut-off^a^	Se (%)	Sp (%)	PPV (%)	NPV (%)
Fast PSTR (> 0.73)	0.749	0.670–0.827	p15 (> 0.66)	100%	30.3%	58.3%	100%
			p20 (> 0.68)	97.3%	38.2%	60.5%	93.5%
			p25 (> 0.69)	91.9%	42.1%	60.7%	84.2%
			p30 (> 0.70)	87.8%	47.4%	61.9%	80.0%

*PSTR* peritoneal solute transfer rate, *ePSTR* estimated peritoneal solute transfer rate, *AUC* area under the curve, *95% CI* 95% confidence interval, *Se* sensitivity, *Sp* specificity, *PPV* positive predictive value, *NPV* negative predictive value

^a^Cut-offs are constructed as percentiles of ePSTR distribution. Thus, p15, p20, p25 and p30 corresponds to the 15th, 20th, 25th and 30th percentiles of ePSTR distribution, respectively

## Discussion

In this study, we described predictors of peritoneal solute transfer rate in incident patients recently starting PD, and developed an equation using readily available clinical parameters to predict transport status without relying on dialysate sampling. Although offering overall moderate performance that can not replace formal peritoneal equilibration test assessment in every clinical setting, this equation allows screening of incident patients starting PD to reliably rule out fast transporters.

It has long been observed that inter-individual variability in peritoneal solute transfer rate is high at dialysis initiation. In the present study, we report a mean peritoneal solute transfer rate value of 0.73 ± 0.13. In prior cohorts including patients from Oceania, the US and the UK, mean peritoneal solute transfer rate was slightly lower, while an Italian group reported a value strictly identical to ours [9, 12–14]. In comparing those numbers, one must bear in mind that variability could arise not only from inherent clinical differences between various studied populations but also from the lack of strict analytical standardization of peritoneal equilibration test assessment [3, 12, 13]. Predictors of peritoneal solute transfer rate at dialysis initiation are still poorly understood with very heterogeneous results among main studies [9, 12, 13, 15]. In our cohort, gender, serum albumin and serum sodium were the three factors with the highest effect size on peritoneal solute transfer rate at dialysis initiation. Specifically, fast transport was associated with male gender as well as lower albumin and sodium serum levels. The gender effect on peritoneal solute transfer rate is well documented and is generally thought to be explained by the relative larger size of men as compared to women as the 3-pore model predicts that transport rate increases when a larger membrane surface area is in contact with the dialysate [12, 13, 15]. In keeping with this hypothesis, the gender effect tended to disappear when accounting for body surface area in prior reports [9, 13]. Conversely however, we observed that male gender remained a strong determinant of fast transport while accounting for body surface area as well as other confounders, potentially suggesting an association with the functional vascular peritoneal area in addition to the anatomic membrane area. Lower serum albumin was previously associated with higher transport rate and has been thought to mediate the relationship between peritoneal solute transfer rate and clinical outcomes, as a global marker of frailty [9, 13]. Peritoneal protein loss could explain the low serum albumin in fast transporters. However, an association between peritoneal solute transfer rate and albumin has been noted prior to dialysis initiation [16]. Alternatively, underlying chronic inflammation could also explain both low albumin and fast transport rate,but, then again, most studies failed to identify a relationship between peritoneal solute transfer rate and inflammatory parameters [17, 18]. Our findings support a direct link between transport status and albumin levels as this relationship remained highly significant despite adjusting for comorbidities and C-reactive protein. Finally, while not previously described in prior publications, we also found that fast transporters had lower serum sodium. An increase in intra-vascular free water is the most likely explanation to this phenomenon as it was previously shown that fast transport allowed relatively more sodium to be removed as compared to water in those patients [19]. This is also corroborated by a positive association between extracellular water/total body water and peritoneal solute transfer rate in our cohort. Conversely, slow peritoneal transport could magnify sodium sieving and hamper sodium removal when short dwells are prescribed. Lastly, the influence of PD mode (CAPD vs APD) must be remembered as dialytic sodium removal was shown to be less efficient relative to water removal with APD as compared to CAPD [19]. This would support the hypothesis that shorter dwell used in APD favors sodium sieving and water removal through aquaporins that cannot be compensated by later convective transfer of sodium. The present work was however not designed to confirm those physiological hypotheses and the interested reader might refer to a previous publication [19].

The main finding of our study is the potential clinical application of an equation based on readily available clinical variables to predict transport status in incident PD patients without relying on dialysate sampling. Our equation is obviously not intended to universally replace formal peritoneal equilibration test assessment as it allows estimation of a single metric (peritoneal solute transfer rate) with moderate accuracy only. Our model could thus only explain 18.4% of peritoneal solute transfer rate variance observed in the validation group. We believe however that it could have clinically meaningful applications as detection of fast peritoneal solute transfer rate is important to guide PD prescription in daily practice [3]. Using our equation, an estimated peritoneal solute transfer rate cut-off above the 15th (> 0.66), 20th (> 0.68), 25th (> 0.69) and 30th (> 0.70) percentiles could rule out fast peritoneal solute transfer rate (> 0.73) with negative predictive values of 100%, 93.5%, 84.2% and 80.0%, respectively. That performance allows exclusion of fast peritoneal solute transfer rate at dialysis initiation in a significant proportion of patients with high clinical certainty. Such information could potentially be used in incident patients to guide initial PD prescription prior to peritoneal equilibration test assessment as well as later during follow-up to ensure adequacy of dialytic therapy.

Peritoneal solute transfer rate values show significant regional discrepancies owing to differences in populations as well as procedural and laboratory standardization [3, 9, 12–14]. However, peritoneal solute transfer rate is normally distributed with a very reproducible standard deviation across various populations [3]. It is also reproducible within a given individual with a coefficient of variation < 10% within a month of testing [3]. Consequently, we chose to develop a predictive model based on relative peritoneal solute transfer rate values, based on mean and percentiles thus potentially allowing direct application to other populations with different absolute peritoneal solute transfer rate values. It is also worth noting that our model only uses four readily available variables (gender, race, serum albumin and serum sodium) to achieve discrimination. As such, it could be potentially implemented in many clinical settings, even those with particularly low resources. Using our equation, negative predictive values (rule out) were notably higher than positive predictive values (rule in) to detect fast peritoneal solute transfer rate. This is however expected by design as we deliberately chose cut-offs that would allow exclusion of fast peritoneal solute transfer rate, a result that would have the most impact regarding clinical decisions at dialysis initiation. While prior publications described various equations aiming at estimating residual kidney function without relying on 24-h urine collection, we could not find comparable studies focusing on peritoneal solute transfer rate estimation [20–22]. Our results could thus not be compared with those of other groups and should rather constitute a springboard to future research.

Readers should bear in mind several limitations when interpreting our findings. First, our study is cross-sectional in nature and longitudinal follow-up was not available in our cohort. Consequently, our findings would apply to incident patients only and performance of our equation to detect later changes in peritoneal solute transfer rate during follow-up could not be assessed. This aspect should be addressed before considering application in a real-life setting. Second, similar to most publications focusing on non-invasive predictive equations, we used an internal validation procedure that should be externally validated before considering pragmatic application [20–22]. Overfitting of our model was however prevented by randomly splitting our cohort into two distinct groups of satisfactory sizes. Moreover, in agreement with the latest guidelines, we used relative peritoneal solute transfer rate measurements (mean and percentiles) and not absolute values. We could thus rely on the reproducibility of peritoneal solute transfer rate distribution across populations without using center-specific peritoneal solute transfer rate absolute values. Finally, our equation includes self-reported race that has been criticized as a sociopolitical construct mediating the effect of structural racism in estimating glomerular filtration rate [23]. In the present setting however, we believe that this should be tempered as classifying solute transfer rate in PD patients is obviously not as sociologically delicate as the estimation of kidney function.

## Conclusion

We developed an equation to predict fast peritoneal solute transfer rate in incident PD patients without requiring dialysate sampling based on a limited set of simple clinical variables. While our model is not intended to replace formal peritoneal equilibration test assessment in every clinical situation, it allows ruling out fast transport in a significant proportion of patients with a high degree of confidence. It could thus prove useful in the daily clinical care of PD patients in guiding both initial prescription as well as later adaptations of dialytic regimen, particularly in low-resource settings. Further prospective studies are required to determine whether such an equation could improve patient care in a real-world setting.

## Data Availability

Available from corresponding author upon reasonable request.

## Abbreviations

PD: Peritoneal dialysis
CAPD: Continuous ambulatory peritoneal dialysis
APD: Automated peritoneal dialysis
PET: Peritoneal equilibration test
PSTR: Peritoneal solute transfer rate
ePSTR: Estimated solute transfer rate
ESKD: End-stage kidney disease
SD: Standard deviation
IQR: Interquartile range
BP: Blood pressure
BSA: Body surface area
BMI: Body mass index
SBP: Systolic blood pressure
DBP: Diastolic blood pressure
ECW: Extracellular water
TBW: Total body water
CRP: C reactive protein
PTH: Parathyroid hormone
AUC: Area under the curve
CI: Confidence interval
Se: Sensitivity
Sp: Specificity
PPV: Positive predictive value
NPV: Negative predictive value

## References

[1] Coester AM, Smit W, Struijk DG, Krediet RT (2009). Peritoneal function in clinical practice: the importance of follow-up and its measurement in patients. Recommendations for patient information and measurement of peritoneal function. NDT Plus.

[2] Van Biesen W, Heimburger O, Krediet R (2010). Evaluation of peritoneal membrane characteristics: clinical advice for prescription management by the ERBP working group. Nephrol Dial Transplant.

[3] Morelle J, Stachowska-Pietka J, Öberg C (2021). ISPD recommendations for the evaluation of peritoneal membrane dysfunction in adults: classification, measurement, interpretation and rationale for intervention. Perit Dial Int.

[4] Brimble KS, Walker M, Margetts PJ (2006). Meta-analysis: peritoneal membrane transport, mortality, and technique failure in peritoneal dialysis. J Am Soc Nephrol.

[5] Badve SV, Hawley CM, McDonald SP (2008). Automated and continuous ambulatory peritoneal dialysis have similar outcomes. Kidney Int.

[6] Yang X, Fang W, Bargman JM, Oreopoulos DG (2008). High peritoneal permeability is not associated with higher mortality or technique failure in patients on automated peritoneal dialysis. Perit Dial Int.

[7] Gu J, Bai E, Ge C (2022). Peritoneal equilibration testing: your questions answered. Perit Dial Int J Int Soc Perit Dial.

[8] Rodríguez-Carmona A, Pérez-Fontán M (2013). ¿Es útil la cinética peritoneal en la práctica clínica? En contra. Nefrologia.

[9] Rumpsfeld M, McDonald SP, Purdie DM (2004). Predictors of baseline peritoneal transport status in Australian and New Zealand peritoneal dialysis patients. Am J Kidney Dis.

[10] Larpent L, Verger C (1990). The need for using an enzymatic colorimetric assay in creatinine determination of peritoneal dialysis solutions. Perit Dial Int.

[11] Davenport A (2013). Effect of intra-abdominal dialysate on bioimpedance-derived fluid volume status and body composition measurements in peritoneal dialysis patients. Perit Dial Int.

[12] Mehrotra R, Ravel V, Streja E (2015). Peritoneal equilibration test and patient outcomes. Clin J Am Soc Nephrol.

[13] Davies SJ (2004). Longitudinal relationship between solute transport and ultrafiltration capacity in peritoneal dialysis patients. Kidney Int.

[14] La Milia V, Cabiddu G, Virga G (2017). Peritoneal equilibration test reference values using a 3.86% glucose solution during the first year of peritoneal dialysis: results of a multicenter study of a large patient population. Perit Dial Int.

[15] Lambie M, Chess J, Donovan KL (2013). Independent effects of systemic and peritoneal inflammation on peritoneal dialysis survival. J Am Soc Nephrol.

[16] Margetts PJ, McMullin JP, Rabbat CG, Churchill DN (2000). Peritoneal membrane transport and hypoalbuminemia: cause or effect?. Perit Dial Int.

[17] Herzig KA, Purdie DM, Chang W (2001). Is C-reactive protein a useful predictor of outcome in peritoneal dialysis patients?. J Am Soc Nephrol.

[18] Wang T, Heimbürger O, Cheng HH (1999). Does a high peritoneal transport rate reflect a state of chronic inflammation?. Perit Dial Int.

[19] Jaques DA, Davenport A (2021). Characterization of sodium removal to ultrafiltration volume in a peritoneal dialysis outpatient cohort. Clin Kidney J.

[20] Jaques DA, Davenport A (2021). Serum β2-microglobulin as a predictor of residual kidney function in peritoneal dialysis patients. J Nephrol.

[21] Shafi T, Michels WM, Levey AS (2016). Estimating residual kidney function in dialysis patients without urine collection. Kidney Int.

[22] Wong J, Sridharan S, Berdeprado J (2016). Predicting residual kidney function in hemodialysis patients using serum β-trace protein and β2-microglobulin. Kidney Int.

[23] Eneanya ND, Boulware LE, Tsai J (2022). Health inequities and the inappropriate use of race in nephrology. Nat Rev Nephrol.

